# The Neuroprotective Effects of 17β-Estradiol Pretreatment in a Model of Neonatal Hippocampal Injury Induced by Trimethyltin

**DOI:** 10.3389/fncel.2018.00385

**Published:** 2018-10-26

**Authors:** Elisa Marchese, Valentina Corvino, Valentina Di Maria, Alfredo Furno, Stefano Giannetti, Eleonora Cesari, Paola Lulli, Fabrizio Michetti, Maria Concetta Geloso

**Affiliations:** ^1^Institute of Anatomy and Cell Biology, Università Cattolica del Sacro Cuore, Rome, Italy; ^2^Epilepsy Center, Department of Clinical Sciences, Lund University, Lund, Sweden; ^3^Laboratory of Neuroembryology, Fondazione Santa Lucia, Rome, Italy; ^4^Laboratorio di Biochimica Clinica e Biologia Molecolare, IRCCS Fondazione Policlinico A. Gemelli, Rome, Italy; ^5^Facoltà di Medicina e Chirurgia - IRCCS San Raffaele Scientific Institute, Università Vita-Salute San Raffaele, Milan, Italy

**Keywords:** hippocampus, trimethyltin, post-natal development, neuroprotection, estrogen, neuroinflammation

## Abstract

Hippocampal dysfunction plays a central role in neurodevelopmental disorders, resulting in severe impairment of cognitive abilities, including memory and learning. On this basis, developmental studies represent an important tool both to understanding the cellular and molecular phenomena underlying early hippocampal damage and to study possible therapeutic interventions, that may modify the progression of neuronal death. Given the modulatory role played by 17β-estradiol (E2) on hippocampal functions and its neuroprotective properties, the present study investigates the effects of pretreatment with E2 in a model of neonatal hippocampal injury obtained by trimethyltin (TMT) administration, characterized by neuronal loss in CA1 and CA3 subfields and astroglial and microglial activation. At post-natal days (P)5 and P6 animals received E2 administration (0.2 mg/kg/die i.p.) or vehicle. At P7 they received a single dose of TMT (6.5 mg/kg i.p.) and were sacrificed 72 h (P10) or 7 days after TMT treatment (P14). Our findings indicate that pretreatment with E2 exerts a protective effect against hippocampal damage induced by TMT administration early in development, reducing the extent of neuronal death in the CA1 subfield, inducing the activation of genes involved in neuroprotection, lowering the neuroinflammatory response and restoring neuropeptide Y- and parvalbumin- expression, which is impaired in the early phases of TMT-induced damage. Our data support the efficacy of estrogen-based neuroprotective approaches to counteract early occurring hippocampal damage in the developing hippocampus.

## Introduction

Because of the central role exerted by the hippocampus in learning and memory, early-life hippocampal dysfunction is believed to underlie many neuropsychiatric and neurodevelopmental disorders, accompanied by severe and permanent cognitive impairment and frequently associated with comorbid pathologies, including epilepsy (DeLong, 1992; Beauregard and Bachevalier, 1996; DeLong and Heinz, 1997; Fisher et al., 1998; de Haan et al., 2006; Bartsch, 2012).

It is well known that the developmental changes occurring in the immature brain are susceptible to particular patterns of vulnerability (Johnston, 1995). The hippocampus, in particular, is vulnerable to a number of insults, such as traumatic brain injury, inflammation, hypoxia, and seizures (Auer et al., 1984; Schmidt-Kastner and Freund, 1991; Tasker et al., 1992; Atkins, 2011; Small et al., 2011; Förster et al., 2012), often leading to a permanent and selective lesion, indicated as hippocampal sclerosis. This is a complex neuropathological entity characterized by severe neuronal loss and gliosis preferentially affecting the CA1 and CA3 subfields (Sloviter, 2005), which frequently represents the neuropathological substrate of temporal lobe epilepsy (Blümcke et al., 2013) and/or cognitive impairment (Oddo et al., 2003), including learning of social skills and language abilities (DeLong and Heinz, 1997).

In light of the above, studies of the cellular and molecular phenomena underlying early hippocampal damage, as well as of possible neuroprotective strategies aimed at counteracting the progression of neuronal loss, could be particularly relevant. A useful tool in this regard is represented by the neonatal rodent hippocampus, since its development and full maturation take place postnatally (Altman and Bayer, 1990), thus modeling the perinatal/neonatal period in humans (Avishai-Eliner et al., 2002). In this respect, the trimethyltin (TMT)-induced hippocampal damage is regarded as a useful model to explore how progressive neurodegenerative processes affect neuronal and glial responses and influence related molecular pathways in both adult and developing rats (Miller and O’Callaghan, 1984; Balaban et al., 1988; Koczyk, 1996; Geloso et al., 1998, 2011; Corvino et al., 2013; Lattanzi et al., 2013; Toesca et al., 2016). In addition, it is also widely used to investigate possible neuroprotective strategies, as indicated by many findings (Shin et al., 2011; Corvino et al., 2012, 2013, 2015).

During postnatal development, in rats, TMT administration causes extensive and progressive subacute neuronal loss, developing over 3 weeks, mainly involving the CA1 and CA3 subfields (Geloso et al., 1998, 2011; Toesca et al., 2016) and associated with memory and learning impairment (Stanton et al., 1991). In line with other models of hippocampal damage resulting in hippocampal sclerosis (Bouilleret et al., 1999; Duveau et al., 2011), loss of pyramidal neurons is associated with alterations in intra-hippocampal circuitry, as indicated by impaired post-natal dentate neurogenesis (Toesca et al., 2016) and changes in the size of some interneuron subpopulations. In particular, a reduced number of parvalbumin (PV)-expressing interneurons in the CA3 subfield (Geloso et al., 1998), together with an altered pattern of reelin expression, have been described (Toesca et al., 2016). These findings suggest that TMT administration during development induces a dysfunction of the GABAergic system, as in adult animals (Koczyk, 1996). In this regard, much evidence supports the role of 17β-estradiol (E2) as a neuroprotective agent in adult life (Garcia-Segura et al., 2001; Melcangi and Panzica, 2006; Spencer et al., 2008) and, although conflicting results have been reported (McCarthy, 2008, 2009), many observations also support the neuroprotective effects of E2 administration in different neurodevelopmental models of brain injury. In physiological conditions estrogens play a relevant role in the development and maturation of specific regions of the central nervous system (CNS), including the hippocampus (McCarthy, 2008, 2009). Through a complex interplay involving both nuclear and non-nuclear receptors, E2 regulates physiologically occurring cell death, involving the classical Bax/Bcl-2 signaling pathway (Forger et al., 2004), influences sexual dimorphism among brain regions (McCarthy, 2008, 2009), as well as synaptic plasticity and neurotransmission, including maturation of GABAergic transmission at the hippocampal level (Wójtowicz and Mozrzymas, 2010). In experimental models of brain injury, pretreatment with E2 protects newborn rats from hypoxia/ischemia (Nuñez et al., 2007), female neonatal rats from kainic-induced dentate gyrus (DG) granule cell loss (Hilton et al., 2004), and neonatal rats subjected to excitotoxic insult mimicking white matter and cortical damages frequently observed in very preterm infants (Pansiot et al., 2016, 2017). E2 is not only able to counteract neuronal death, but also exerts neuroprotection by enhancing neural plasticity, particularly at the hippocampal level (Brinton, 2009). Numerous studies provide evidence that E2 exerts neuroprotective effects by inducing phenotypic changes in the size of different GABAergic subpopulations, in particular enhancing neuropeptide Y (NPY) expression (Nakamura and McEwen, 2005; Corvino et al., 2015) and modulating the size of PV-positive interneuron subpopulation (Rewal et al., 2005; Macrì et al., 2010). Because of the relevance of hippocampal dysfunction and interneuron imbalance in neurodevelopmental and neuropsychiatric diseases, in the present study we investigated the effects of pretreatment with E2 on different aspects related to TMT-induced hippocampal injury in neonatal rats, including neuronal death, neuroinflammation and interneuron impairment, with the aim of providing an exhaustive description of cellular and molecular events related to possible neuroprotective approaches in early hippocampal damage.

## Materials and Methods

### Animal Treatment and Experimental Design

Newborn Wistar rats coming from twelve different litters were used. On the day of birth, litters were culled randomly to preserve eight pups per litter, and pups were randomly assigned to different treatment groups. At post-natal day (P)5, in concomitance with the expression of estrogen receptors (Solum and Handa, 2001), animals were divided into males (M) and females (F), based on anogenital distance. Each group was further divided into two experimental groups (M+oil, F+oil, M+E2, F+E2) and treated with E2-3 benzoate (E2) (Sigma, St Louis, MO, United States) (0.2 mg/kg intraperitoneal – i.p.) or vehicle (sesame oil). The dose of E2 was chosen in accordance with beneficial effects described in our previous study in TMT-treated adult rats (Corvino et al., 2015) and with previously reported early treatment with E2 associated with neuroprotective effects in neonatal rats (Pansiot et al., 2016). The same E2/vehicle dose was administered on post-treatment day 1 (P6). These time points were chosen in order to maximize the effects of the treatment, as they correspond to the critical perinatal period during which the developing hippocampus expresses highest levels of estrogen receptors (O’Keefe and Handa, 1990). In addition, this schedule can be useful to support E2-mediated protective effects on PV-expressing interneurons, which are known to be affected by administration of TMT (Geloso et al., 1998). It is well known that PV-immunoreactivity appears postnatally between P4 and P7 (Danglot et al., 2006) and a correlation between administration of E2 and PV-expression has been hypothesized (Rewal et al., 2005; Macrì et al., 2010; Corvino et al., 2015).

After treatment, the pups were returned to the dams, which were kept on a 12 h light/dark cycle with *ad libitum* access to food and water. Sensitivity to TMT is age-dependent and, in rats, develops only in concomitance with the functional maturation (after P5) of pyramidal neurons in the Cornu Ammonis (CA) (Miller and O’Callaghan, 1984), which are the main target of the neurotoxicant (Geloso et al., 2011). Accordingly, at P7, each group was further divided into two groups and received a single i.p. injection of either saline (CTRL groups) or TMT chloride (Sigma, St. Louis, MO, United States) dissolved in saline at a dose of 6.5 mg/kg body weight in a volume of 1 ml/kg body weight (TMT-treated groups). This dosage was chosen because lower doses, previously used in the same experimental conditions by our group (Geloso et al., 1998; Toesca et al., 2016), failed to induce hippocampal damage, possibly on account of differences in colonies. The following experimental groups were examined: M-CTRL+oil; M-CTRL+E2; F-CTRL+oil; F-CTRL+E2; M-TMT+oil; M-TMT+E2; F-TMT+oil; F-TMT+E2.

Animals intended for immunohistochemistry were sacrificed at P14, when neuronal damage is clearly detectable and neurodegeneration is still ongoing (Balaban et al., 1988; Geloso et al., 1998; Toesca et al., 2016). Rat pups intended for quantitative real-time PCR (qPCR) analysis were sacrificed at two different time points: P10 (that is 72 h after TMT treatment), in order to detect early events associated with pretreatment with E2, and P14, to investigate long-lasting effects related to E2 administration.

### Gene Expression Analysis

Animals intended for qPCR were sacrificed by decapitation after deep anesthesia (ketamine 80 mg/kg i.m. and medetomidine 1 mg/kg i.p.) 3 or 7 days after TMT/saline treatment (*n* = 4/each experimental group). The hippocampi were bilaterally removed and processed for total RNA extraction.

Total RNA was isolated using Trizol reagent (Invitrogen, Carlsbad, CA, United States) and purified using the RNeasy Mini Elute Cleanup Kit (Qiagen, Valencia, CA, United States), according to the manufacturer’s instructions. RNA isolation, reverse transcription and qPCR were carried out as previously described (Corvino et al., 2012, 2014, 2015).

Sequence-specific oligonucleotide primers were used to amplify the following genes (Supplementary Table 1): B-cell leukemia/lymphoma 2-like protein (*Bcl-2*), brain-derived neurotrophic factor (*Bdnf*), neurotrophic tyrosine kinase receptor type 2 (*Ntrk2* also known as *TrkB*), *Npy*, parvalbumin (*Pva*), interleukin 1 beta (*Il1b*), tumor necrosis factor (*Tnf*), interleukin 6 (*Il6*), chitinase 3-like 3 (*Chi3l3* also known as *Ym1*), interleukin 10 (*Il10*), interleukin 4 (*Il4*), S100B (*s100b*) and the long form of aromatase (*Cyp19a1*) (Tabatadze et al., 2014). The oligonucleotide primers were designed using Primer 3 software^1^.

The 2^-ΔΔ*C*_*T*_^ method (Livak and Schmittgen, 2001) was applied to calculate fold differences (fold change, FC) in gene expression, using the gene beta-actin (*Actb*) as the housekeeping reference for data normalization.

### Immunocytochemistry

Rats from the eight experimental groups intended for histology and immunocytochemistry were sacrificed 7 days after TMT or saline administration. Under deep anesthesia (ketamine 80 mg/kg and medetomidine 1 mg/kg i.p.), the animals were perfused with 4% phosphate-buffered saline (PBS) paraformaldehyde; the brains were removed from the skull, cryopreserved in 30% sucrose and then cut on a freezing microtome to obtain 40 μm serial sagittal sections, from 0.9 to 3.4 mm lateral to the midline, according to Paxinos and Watson’s atlas (Paxinos and Watson, 1986). Every sixth section was stained with Nissl or Fluoro Jade-C (FJ) (Chemicon, Temecula, CA, United States) staining to evaluate neuronal degeneration, or by immunocytochemistry to detect of the following antigens: PV and NPY, markers of interneuron subpopulations, Aggrecan (AG), marker of perineuronal net (PNN), Iba1, microglial marker, and CD68, marker of activated microglia.

Sections were incubated overnight with rabbit polyclonal anti-NPY- (AbCam, Cambridge, United Kingdom; 1:2000) and rabbit polyclonal anti-PV- (AbCam, Cambridge, United Kingdom; 1:2000) antibodies. NPY reaction was developed using the avidin-biotin peroxidase complex (ABC method, Vector, Burlingame, CA, United States) and 3,3′-diaminobenzidine (Sigma, St. Louis, MO, United States) as a chromogen. PV reaction was developed using goat anti-rabbit FITC (Vector, Burlingame, CA, United States 1:200, 1 h at room temperature).

Co-expression of PV and AG or Iba1 and CD68 was identified by fluorescent double-labeling using mouse monoclonal anti-PV- (Swant, Bellinzona, Switzerland; 1:10000) and rabbit polyclonal anti-AG- (Chemicon, Temecula, CA, United States, 1:1000) antibodies or rabbit polyclonal anti-Iba1- (Wako, Richmond, VA, United States; 1:1000) and mouse monoclonal anti-CD68- (Bio-Rad/AbD Serotec, Oxford, United Kingdom; 1:100) antibodies and revealed using secondary FITC-conjugated antibody (goat anti-rabbit- or goat anti-mouse- FITC, Vector, Burlingame, CA, United States 1:200, 1 h at room temperature) and secondary cyanine-3-conjugated antibody (donkey anti-mouse Cy3 or donkey anti-rabbit Cy3 1:400, 1 h at room temperature) (Jackson Immunoresearch Laboratories, West Grove, PA, United States).

Negative controls were processed in the absence of the primary antibodies. Confocal laser scanning microscope (LSM 510 META, Zeiss, Oberkochen, Germany) was employed to analyze the co-localization of the markers investigated.

### Quantitative Analysis

#### Stereological Cell Counts

Unbiased determination of the total number of FJ-, PV- or NPY- positive neurons in the regions of interests (ROIs), was obtained using a design-based stereological method employing the Stereo Investigator System (Stereo Investigator software, Version 9, MicroBrightField Europe, Magdeburg, Germany), essentially as previously described (Corvino et al., 2012, 2015).

Briefly, a stack of MAC 6000 controller modules (MBF Bioscience, Williston VT, United States) was configured to interface with a Nikon Eclipse 80i microscope with a motorized stage and a digital color camera (MBF Bioscience q imaging) with a Pentium II PC workstation.

FJ- stained degenerating neurons were quantified in the CA1 and CA3 pyramidal layers of TMT-treated groups (F-TMT *n* = 6; M-TMT+oil *n* = 4; F-TMT+E2 *n* = 7; M-TMT+E2 *n* = 4). A three-dimensional optical dissector counting probe (x, y, z dimension of 100 μm × 150 μm × 10 μm, respectively) was applied to a systematic random sample of sites in the ROI (magnification = 40×).

Since stereological approach requires an average of one or two cells in the sampling area, the estimate of PV- and NPY-immunoreactive (IR) neurons was performed only in selected hippocampal layers.

In particular, PV-IR interneurons were quantified only in the *stratum oriens* and in the pyramidal layer of the CA1 subfield, in the pyramidal layer of the CA3 subfield and in the granular layer of the DG (Andressen et al., 1993) of the eight experimental groups (F-CTRL+oil *n* = 6; M-CTRL+oil *n* = 5; F-CTRL+E2 *n* = 5; M-CTRL+E2 *n* = 4; F-TMT+oil *n* = 5; M-TMT+oil *n* = 4; F-TMT+E2 *n* = 4; M-TMT+E2 *n* = 4). A three-dimensional optical dissector counting probe (counting probe: x, y, z dimension of 200 μm × 250 μm × 10 μm, respectively) was applied to a systematic random sample of sites in the region of interest at a magnification of 20×.

For the same reason, quantitative analysis of NPY-IR interneurons was performed only in the *stratum oriens* and in the pyramidal layer of the CA1 subfield, in the hilus and in the granular layer of the DG, in line with previous observations (Deller and Leranth, 1990; Sperk et al., 2007; Corvino et al., 2015) (counting probe: x, y, z dimension of 200 μm × 250 μm × 10 μm, respectively, magnification of 40×) (F-CTRL+oil *n* = 5; M-CTRL+oil *n* = 5; F-CTRL+E2 *n* = 6; M-CTRL+E2 *n* = 4; F-TMT+oil *n* = 5; M-TMT+oil *n* = 5; F-TMT+E2 *n* = 5; M-TMT+E2 *n* = 5).

#### Confocal Microscope Double-Staining Quantitative Analysis

Double-stained PV/AG- (F-CTRL+oil *n* = 3; M-CTRL+oil *n* = 3; F-CTRL+E2 *n* = 4; M-CTRL+E2 *n* = 3; F-TMT+oil *n* = 4; M-TMT+oil *n* = 3; F-TMT+E2 *n* = 3; M-TMT+E2 *n* = 3) and Iba1/CD68- (F-TMT+oil *n* = 3; M-TMT+oil *n* = 3; F-TMT+E2 *n* = 3; M-TMT+E2 *n* = 3) cells were quantified in the eight experimental groups using z-scan confocal microscopy at 40× magnification. The entire length of the CA1 and CA3 pyramidal layers was evaluated through the septo-temporal axis of the hippocampus in 1-in-12 series of sections, as previously described (Corvino et al., 2012, 2014). The count of double-labeled cells was performed manually by an experimenter who was not informed of the group assignment.

The following formula was used to calculate the estimate of total number of IR cells for each marker in each case: *E* = 12Σ*N* (*E* = estimate of the total number of stained cells in each case; Σ*N* = sum of *n* values in the *n* sections considered, 12 indicates that every 12th section was considered). The *N*-value was obtained applying Abercrombie’s correction, *N* = *nt*/(*t* + *D*) (*n* = number of cells counted in each section, *t* = thickness of the section, and *D* = mean diameter of the cells) (Abercrombie, 1946), as previously described (Geloso et al., 2004; Corvino et al., 2012, 2014).

The quantification of double-stained cells was expressed as the percentage of PV/AG- or Iba1/CD68- double-labeled cells in relation, respectively, to the total number of PV-IR or Iba1-IR cells.

#### Quantification of AG Immunofluorescence Intensity

Images from double-stained PV/AG samples were acquired with LSM 510 META confocal laser scanning microscopy system (Zeiss, Oberkochen, Germany) using a 63× oil immersion lens. The confocal pinhole was kept at the minimum (1.0), the gain and the offset were lowered to prevent saturation in the brightest signals. These settings were kept constant for all images. Pooled groups (males + females) were evaluated and six animals from each experimental group (CTRL+oil; CTRL+E2; TMT+oil; TMT+E2) were used for the quantitative analysis (total number of sections/animal = 4, number of double-stained PV/AG cells/section/hippocampal region = 10). Quantitative image analysis was performed using the Java ImageJ image-processing and analysis program (NIH) (Schneider et al., 2012; Jensen, 2013; Slaker et al., 2016). Each image was opened in ImageJ, and screened initially by splitting into color channels (PV-green and AG-red). For each channel, a binary image was created by thresholding to exclude background fluorescence. Thresholding values were kept constant between each image. The Rolling Ball Radius function in ImageJ was used to remove variability in background staining across the image. Each image was then converted to a maximum intensity Z-stack projection image. For the quantification of AG fluorescence intensity in PV+ interneurons, since AG is located around the outside of neurons, a ROI was constructed around the AG label surrounding the soma of individual PV+ cells, excluding proximal dendrites, using the freehand drawing tool in ImageJ. Background intensity was measured in a region adjacent to the cell and subtracted from the intensity measurement. Then AG mean fluorescent pixel intensity and fractional area of ROI were then measured and expressed, respectively, as arbitrary intensity units and as a percentage of the total area examined.

### Statistical Analysis

Three-way ANOVA with TMT and E2 treatments and sex as the main factors or two-way ANOVA with E2 treatments and sex as the main factors were performed for statistical group comparison, as previously described (Geloso et al., 1996, 1998; Corvino et al., 2014). Tukey’s HSD *post hoc* test was used, if pertinent, to compare specific groups’ means, with a significance level of *p* < 0.05. Results are expressed as mean ± SE.

Statistical analysis of gene expression data was performed comparing the ΔCt-values across the replicates using an unpaired *t*-test (*p*-value cut-off = 0.05), as previously described (Corvino et al., 2012, 2014).

## Results

### Estrogen Treatment Protects From TMT-Induced Neuronal Death in Juvenile Rat Hippocampus

To test the possible neuroprotective effect of estrogens in TMT-induced hippocampal neurodegeneration, we injected male and female neonatal rats with E2 before treatment with TMT.

It has been proposed that interactions with the pathway of endogenous estrogens may contribute to the effects of exogenous E2 in the brain (Chamniansawat and Chongthammakun, 2012). As in our previous study (Corvino et al., 2015), to test whether TMT treatment affects local estrogenic pathway, we evaluated, by qPCR analysis performed 3 days after TMT administration, the expression levels of the long form of aromatase (*Cyp19a1*), corresponding to the active form of the enzyme (Tabatadze et al., 2014). Aromatase is responsible for E2 synthesis and is expressed in the hippocampus also during the neonatal period (Lephart et al., 1992; Amateau et al., 2004; Hojo et al., 2004; McCarthy, 2008). No differences in *Cyp19a1* expression levels were present among the experimental groups (*p* > 0.05) (Supplementary Figure 1 and Supplementary Table 1), suggesting that TMT administration in neonatal rats does not impair of the expression of key elements of the estrogenic pathway.

**FIGURE 1:**
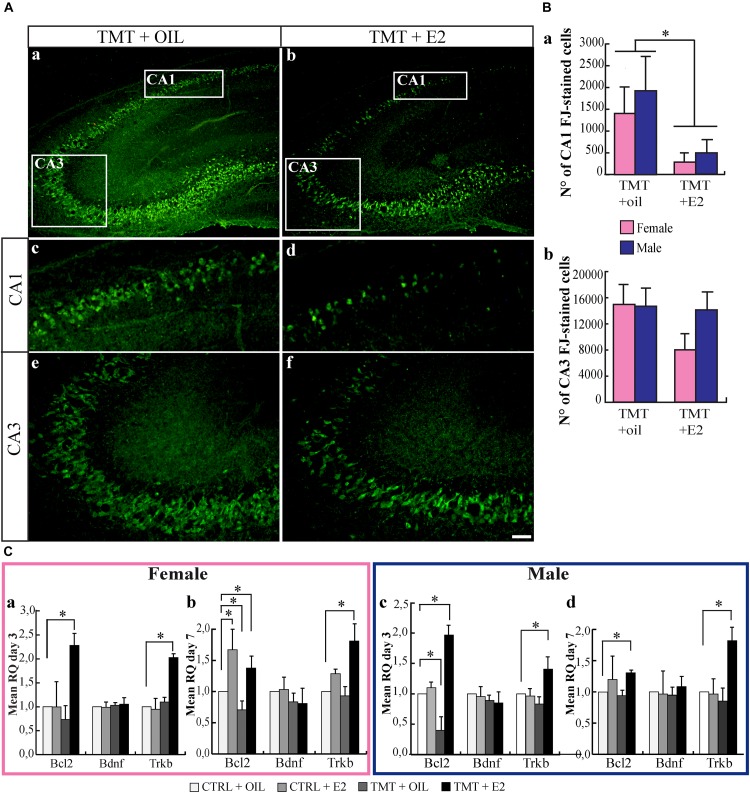
**(A)** 17β-estradiol (E2) reduces Fluoro Jade C (FJ)-stained degenerating neurons in the trimethyltin (TMT)-injured hippocampus. Representative micrographs of FJ-stained hippocampal sagittal sections **(a,b)** from CA1 **(c,d)** and CA3 subfields **(e,f)** of TMT+oil- **(a,c,e)**, and TMT+E2- **(b,d,f)** treated rats. FJ-positive neurons are clearly less numerous in the CA1 pyramidal layer of TMT+E2-treated animals compared with TMT+oil-treated rats. Scale bar: 250 μm in **(a,b)**, 150 μm in **(c–f)**. **(B)** Quantitative analysis of FJ -stained cells in the different experimental groups. Stereological cell counts of FJ-stained cells confirmed that both groups of TMT+E2-treated animals showed fewer degenerating neurons in CA1 compared with TMT+oil-treated groups. Two-way ANOVA revealed a significant effect of E2 treatment in CA1 (*F*_1,17_ = 5.68, Tukey *post hoc p* < 0.05) **(a)**. On the contrary, no significant difference was detectable in the CA3 subfield (*F*_1,17_ = 1.7; *p* > 0.05) **(b)**. No differences related to sex are present (*p* > 0.05 both in CA3 and in CA1). The values are given as means ± SE (^∗^*p* < 0.05) (F-TMT *n* = 6; M-TMT+oil *n* = 4; F-TMT+E2 *n* = 7; M-TMT+E2 *n* = 4). **(C)** Expression levels of genes involved in neuroprotective pathways and their modulation in the hippocampus of TMT-treated rats by E2 administration. Bar graphs represent results of quantitative real-time PCR obtained using the ΔΔCt method for the calculation of relative quantity (RQ), calculated on mean ΔCt across biological replicates, of *Bcl-2*, *Bdnf* and *trkB*, 3 **(a,c)** and 7 **(b,d)** days after TMT treatment both in female **(a,b)** and male **(c,d)** rats (^∗^*p* < 0.05) (*n* = 4/each experimental group).

Nissl-stained hippocampal sections from animals sacrificed 7 days after TMT or saline treatment were analyzed, to assess the features and the extent of the TMT-induced hippocampal damage. In line with previous observations (Geloso et al., 1998), TMT caused significant neuronal loss, involving mainly the pyramidal layer of the CA1 and CA3 subfields of both male and female rats (Supplementary Figure 2). Consistently, many FJ-stained degenerating neurons were present in the CA1 and CA3 subfields of all TMT-treated groups, selectively localized in the pyramidal layer (Figure 1A), while no stained neurons were detectable in the hippocampi of the CTRL groups (not shown). Remarkably, stereological cell counts of FJ-stained cells showed that the pretreatment with E2 significantly reduced neuronal loss in the CA1 subfield of TMT+E2-treated male and female animals compared with TMT+oil-treated groups (Figure 1Ba), with no sex-specific effects (*p* > 0.05). In contrast, no significant difference was detectable in the CA3 subfield (*p* > 0.05), even though a trend of E2-induced neuronal protection was observed in females (Figure 1Bb).

At the molecular level, our data indicate that TMT administration induces changes in *bcl-2* expression, with some sex-related differences. In particular, in males, it causes a downregulation of *bcl-2* expression, which is early (3 days after TMT administration) and transient, with levels not significantly different from CTRL animals at the second time point explored (*p* > 0.05) (Figures 1Cc,d). In females, however, the same phenomenon occurs later (7 days after intoxication) (Figures 1Ca,b).

E2 was proposed to induce the expression of factors that counteract neuronal death, including the anti-apoptotic protein Bcl-2, the neurotrophin BDNF and its corresponding receptor TrkB (McCarthy, 2008). Accordingly, we observed an early and long-lasting upregulation of *bcl-2* and *trkB* mRNA expression by E2 in both female (Figures 1Ca,b) and male (Figures 1Cc,d) TMT-treated animals compared with controls (CTRL+oil; *p* < 0.05) (Supplementary Table 1). A significant *bcl-2* upregulation was also detectable in CTRL+E2 female rat pups at the later time point (Figure 1Cb; *p* > 0.05).

By contrast, no modulation of *Bdnf* expression was evident among groups at either time points after TMT treatment (*p* > 0.05) (Figure 1C).

These results suggest that E2 protects from TMT-induced neuronal degeneration through transcriptional activation of specific protective factors in the developing hippocampus.

### Estrogen Treatment Reduces Neuroinflammatory Pathways in the TMT-Treated Juvenile Rat Hippocampus

**FIGURE 2:**
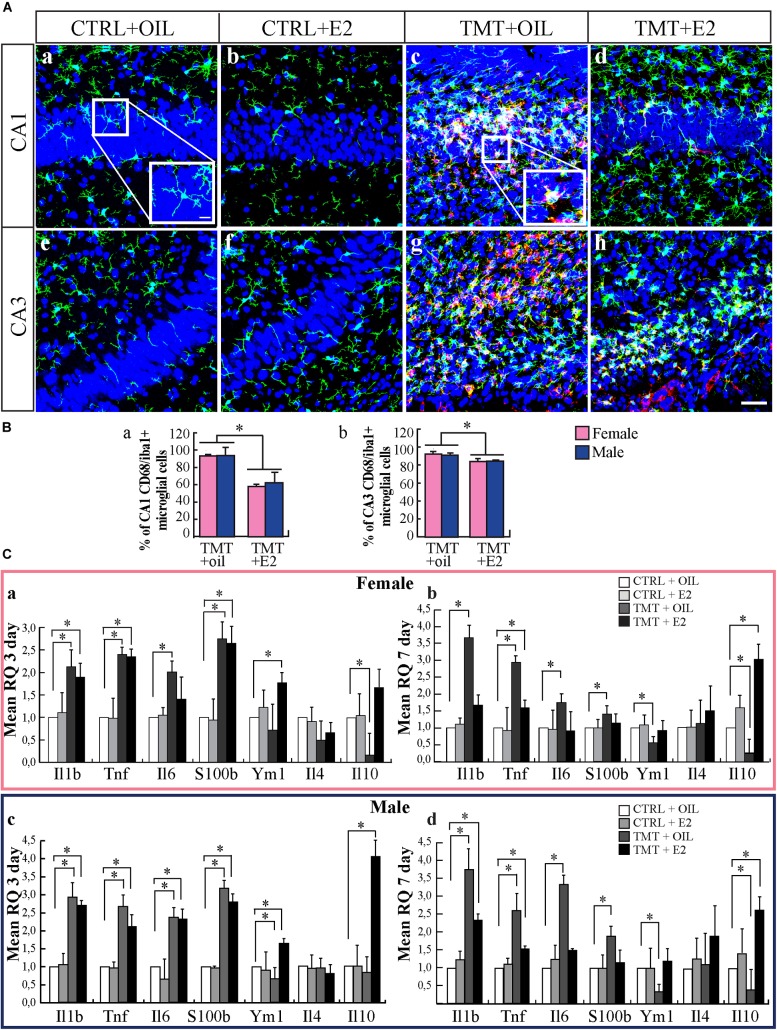
17β-estradiol (E2) reduces microglial activation during trimethyltin (TMT)-induced hippocampal damage. **(A)** Representative confocal microscopy micrographs of CA1 **(a–d)** and CA3 **(e–h)** hippocampal subfields from CTRL+oil- **(a,e)**, CTRL+E2- **(b,f)**, TMT+oil- **(c,g)**, and TMT+E2- **(d,h)** treated animals double-labeled for Iba1 (*green*), CD68 (*red*) and counterstained with the nuclear dye TO-PRO (*blue*). Larger Iba1-immunoreactive cell bodies, exhibiting thicker and shortened processes are evident in the hippocampi of TMT-treated animals (enlarged image included in the box in **c**) compared with CTRL (enlarged image included in the box in **a**). In addition, a reduced expression of activated microglia double-stained for Iba1/CD68 is evident in the TMT+E2-treated group **(d,h)**. Scale bar: 60 μm **(a–h)**. Scale bar: 10 μm (boxes in **a** and **c**). **(B)** Bar graphs indicating the percentage of Iba1/CD68 double-stained cells in CA1 and CA3 pyramidal layers in the different experimental groups. Quantitative analysis of the percentage of double-stained Iba1/CD68 cells on the total number of Iba1-positive cells, followed by two-way ANOVA, reveals a significant effect of E2 treatment in all subfields of the CA region (CA1: *F*_1,8_ = 17.1, *p* < 0.05; CA3: *F*_1,8_ = 8.02, *p* < 0.05). In CA1 **(a)** and CA3 **(b)** regions, a significantly lower percentage of Iba1/CD68 double-stained cells is evident in the hippocampi of both TMT+E2-treated groups (F-TMT+E2 and M-TMT+E2) compared with TMT+oil-treated animals (F-TMT+oil and M-TMT+oil), (Tukey’s HSD *p* < 0.05 for both regions). The values are given as mean ± SE (^∗^*p* < 0.05) (*n* = 3 for each experimental group). **(C)** Expression levels of genes encoding for inflammatory cytokines and their modulation in the hippocampus of TMT-treated rats by E2 administration. At the earliest time point (3 days after TMT administration) qPCR results indicate that all TMT-treated groups show a significant increase in pro-inflammatory cytokines *Il1b-*, *Tnf*-, *Il6*-, and *s100b*- expression compared with relative CTRL+oil groups (Student *t*-test: *p* < 0.05) **(a,c)**. In addition, while both groups of TMT+oil-treated animals show a slight, but significant (in the male group) (*p* < 0.05), reduction in the anti-inflammatory cytokine *ym1*, both TMT+E2-treated groups exhibit an upregulation of the same gene (Student *t*-test: *p* < 0.05) **(a,c)**. TMT treatment also induces a significant downregulation of anti-inflammatory gene *Il10* only in female rats while a significant increase in *Il10* expression is observed in M-TMT-treated rats after E2 administration. At the later time point, a persistent upregulation of pro-inflammatory cytokines (*Il1b, Tnf, Il6*, and *s100b*) is still evident in both female **(b)** and male **(d)** TMT+oil-treated rat pups, associated with a significant downregulation of the anti-inflammatory *ym1* and *Il10* (Student *t*-test: *p* < 0.05). Expression levels of pro-inflammatory cytokines comparable to CTRL animals (*p* > 0.05) were detectable in both groups of TMT+E2-treated animals at this time point (*p* < 0.05), although an attenuated but long-lasting upregulation of *Tnf* was still detectable in TMT+E2-treated rats (*p* < 0.05) **(b,d)**, together with an increased expression of *Il1b* levels in the M-TMT+E2-treated (*p* < 0.05) **(d)**; furthermore a significant upregulation of anti-inflammatory gene *Il10* was observed both in female **(b)** and male **(d)** TMT+E2-treated rats while no changes in *Il4* expression levels were present (*n* = 4/each experimental group).

Neuroinflammation is a relevant feature of TMT-induced neurodegeneration (for review, see Geloso et al., 2011; Corvino et al., 2013; Lattanzi et al., 2013). Hippocampal microglia achieve a mature phenotype during the second postnatal week and could be particularly sensitive to activation (Harry, 2013; Kim et al., 2015). By using Iba1 as a marker of microglia, we found that TMT-treated animals showed the presence of larger Iba1-IR cell bodies, exhibiting thicker and shorter processes compared with CTRL groups, mainly localized in the CA1 (Figures 2Aa–d) and CA3 (Figures 2Ae–h) pyramidal layers, in close proximity to degenerating neurons. In addition, many Iba1–IR cells also exhibited a clear immunoreactivity for the microglia activation marker CD68 (Figures 2Ac,d,g,h) (Graeber et al., 1990; Slepko and Levi, 1996; Kingham et al., 1999; Marchese et al., 2017), in contrast to CTRL groups, which showed little or no CD68-immunoreactivity (Figures 2Aa,b,e,f). Pretreatment with E2 reduced the number of activated microglial cells in the main sites of TMT-induced neuronal death, as indicated by quantitative analysis of double-stained Iba1/CD68 cells, which shows a significantly lower percentage of Iba1/CD68 double-stained cells in the hippocampi of TMT+E2-treated groups compared with TMT+oil-treated animals, in both the CA1 (Figure 2Ba) and CA3 (Figure 2Bb) regions (Tukey’s HSD *p* < 0.05 for both regions).

We also evaluated, by qPCR, the expression levels of genes related to pro-inflammatory cytokines, such as *Il1b, Tnf, Il6*, and *s100b*, as well as to the anti-inflammatory cytokines *Ym1*, *Il10*, and *Il4*.

As expected, TMT administration induced a significant increase in the expression of pro-inflammatory genes (*Il1b*, *Tnf*, *Il6*, and *s100b; p* < 0.05) already evident at 3 days after treatment (Figures 2Ca,c), and still detectable at the later time point (7 days, Figures 2Cb,d). It also caused significant downregulation of *Ym1* and *Il10* expression (*p* < 0.05; Figures 2Cb,d), while no changes in the expression levels of *Il4* were detectable (*p* > 0.05, Figures 2Cb,d).

E2 treatment reduces the expression of the gene encoding for the proinflammatory cytokines *Il6* and *s100b* in both groups of TMT treated animals (Figures 2Ca,b) and is associated with some sex-related differences in the expression profile of some proinflammatory cytokines. In particular, in males, E2 administration is not able to modulate the expression levels of *Tnf* and *Il1b*, which appear to be persistently higher than CTRL animals in both TMT+oil and TMT+E2-treated groups (Figures 2Cc,d). In contrast, in female animals, pretreatment with E2, even though is not able to downregulate *Tnf* expression, prevents the increase of *Il6* at the first time point, and induces a reduction of *Il1b* expression at levels comparable to CTRL animals 7 days after TMT administration (Figures 2Ca,b).

Notably, in both groups E2 was also able to induce an upregulation of the genes encoding for the anti-inflammatory cytokines *Ym1* (*p* < 0.05; Figures 2Ca,c) and *Il10* (*p* < 0.05; Figures 2Ca,c), which may contribute to reduce the burden of TMT-induced inflammation.

Also in this case some slight differences related to sex emerge, since TMT+E2-treated males show an earlier and persistent increase of *Il10* expression levels (3 days after TMT), while in TMT+E2-treated females the upregulation of *Il10* is evident 7 days after intoxication (Figures 2Cb,d). No E2-mediated changes in the expression levels of *Il4* were detectable among groups (*p* > 0.05, Figures 2Cb,d).

Collectively, these observations suggest that E2 limits in time and amplitude the neuroinflammatory response elicited by TMT in neonatal rats.

### E2 Administration Modulates Intra-Hippocampal Circuitry

E2 is a potent modulator of GABAergic transmission (Wójtowicz et al., 2008; Wójtowicz and Mozrzymas, 2010). Moreover, many findings in adult animals suggest a specific effect of E2 administration on the size of PV- and NPY- expressing neuronal subpopulations, that has been considered part of E2 effects on neuroplasticity and interpreted as neuroprotective phenomena (Nakamura and McEwen, 2005; Rewal et al., 2005; Velísková and Velísek, 2007; Wu et al., 2014; Corvino et al., 2015). Therefore, to test whether E2 administration is able to counteract the damage to the GABAergic interneurons induced by TMT administration (Geloso et al., 1998), we explored the possible effects of pretreatment with E2 on TMT-induced changes in PV- and NPY- expressing neuronal subpopulations.

#### NPY-Expressing Interneurons

**FIGURE 3:**
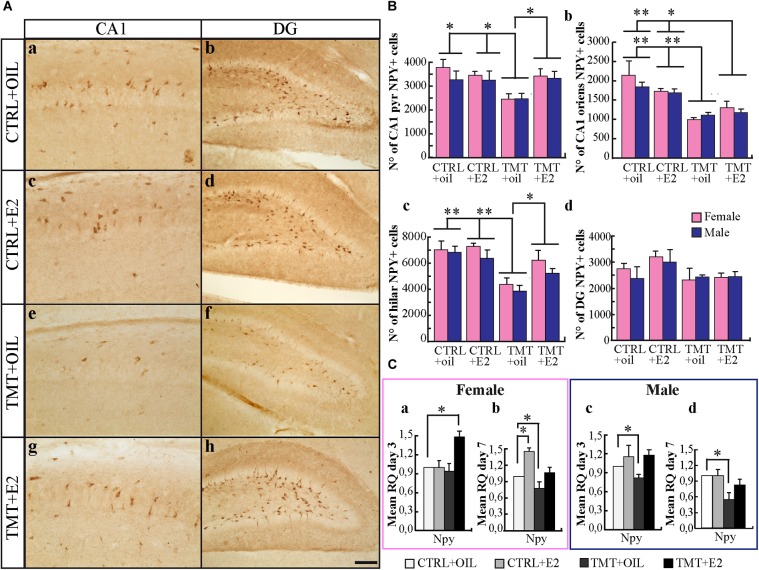
**(A)** Distribution of neuropeptide Y (NPY)-immunostaining in the hippocampus of the different experimental groups. Representative micrographs of NPY-stained hippocampal sagittal sections from CA1 **(a,c,e,g)** and dentate gyrus (DG)/hilus **(b,d,f,h)** hippocampal subfields from CTRL+oil- **(a,b)**, CTRL+17β-estradiol (E2)- **(c,d)**, TMT+oil- **(e,f)**, and TMT+E2- **(g,h)** treated animals. Scale bar: 250 μm in **(a,c,e,g)**; 150 μm in **(b,d,f,h)**. **(B)** Numbers of NPY-positive cells in the hippocampi of the different experimental groups. Quantitative analysis followed by three-way ANOVA performed in the pyramidal layer of the CA1 subfield revealed a significant effect of both TMT treatment (*F*_1,32_ = 6.01, *p* < 0.05) and TMT^∗^E2 interaction (*F*_1,32_ = 6.63, *p* < 0.05); no significant differences were detectable between males and females (*F*_1,32_ = 1.24 *p* > 0.05). Tukey’s HSD *post hoc* test showed that a significantly lower number of NPY-positive neurons was present in TMT+oil-treated animals (F-TMT+oil and M-TMT+oil) compared with all other groups (*p* < 0.05), while no differences were present between TMT+E2-treated animals and CTRL+oil groups (*p* > 0.05) **(a)**. Statistical analysis of cell counts performed in the CA1 *stratum oriens* showed only a significant effect of TMT treatment (*F*_1,32_ = 44.56; *p* < 0.001) and TMT^∗^E2 interaction (*F*_1,32_ = 5.16; *p* < 0.05). Tukey’s *post hoc* test pointed to a significant reduction of NPY-IR neurons in the TMT+oil- and TMT+E2-treated groups compared with CTRL+oil and CTRL+E2 animals (TMT+oil vs. CTRL+oil and CTRL+E2 *p* < 0.001; TMT+E2 vs. CTRL+oil *p* < 0.05; TMT+E2 vs. CTRL+E2 *p* < 0.001) **(b)**. In the hilus, a significant effect of both TMT treatment (*F*_1,32_ = 32.76, *p* < 0.001) and E2 administration (*F*_1,32_ = 4.3, *p* < 0.05) emerged, while sex was not a significant variable (*F*_1,32_ = 3.67, *p* > 0.05). The TMT^∗^E2 interaction was also significant (*F*_1,32_ = 6.21, *p* < 0.05). Tukey’s HSD *post hoc* test showed a significantly lower number of NPY-positive cells in TMT+oil-treated animals (M-TMT+oil and F-TMT+oil) compared with all other groups (TMT+oil vs. CTRL+oil and CTRL+E2 *p* < 0.001; TMT+oil vs. TMT+E2 *p* < 0.05) **(c)**. No significant differences among groups were detectable in the DG (*p* > 0.05) **(d)**. The values are given as mean ± SE (^∗^*p* < 0.05, ^∗∗^*p* < 0.001). (F-CTRL+oil *n* = 5; M-CTRL+oil *n* = 5; F-CTRL+E2 *n* = 6; M-CTRL+E2 *n* = 4; F-TMT+oil *n* = 5; M-TMT+oil *n* = 5; F-TMT+E2 *n* = 5; M-TMT+E2 *n* = 5). **(C)** Expression levels of *npy* gene and its modulation in the hippocampus of TMT-treated rats by E2 administration. Bar graphs represent results of quantitative real-time PCR obtained using the ΔΔCt method for the calculation of relative quantity (RQ) of *npy* gene, 3 **(a,c)** and 7 **(b,d)** days after TMT administration both in female **(a,b)** and male **(c,d)** rats; ^∗^*p* < 0.05, calculated on mean ΔCt across biological replicates. The downregulation of the *npy* gene starting 3 days after intoxication in male TMT-treated animals (M-TMT+oil vs. M-CTRL oil: *p* < 0.05) **(c)** and involving both groups 7 days after treatment (M-TMT+oil vs. M-CTRL oil and F-TMT+oil vs. F-CTRL+oil: *p* < 0.05) is evident **(b,d)**. On the contrary, expression levels comparable to those of CTRL animals were detectable in the TMT+E2-treated groups (M-TMT+E2 vs. M-CTRL+oil and F-TMT+E2 vs. F-CTRL+oil: *p* > 0.05) at the later time point **(b,d)**, which, in the F-TMT+E2 group, is preceded by an earlier and transient upregulation (F-TMT+E2 vs. F-CTRL+oil: *p* < 0.05) **(a)** (*n* = 4/each experimental group).

Light microscope analysis revealed that CTRL+oil and CTRL+E2 young rats exhibited NPY-immunoreactivity in the CA1 pyramidal layer and *stratum oriens*, while only scattered cells were detectable in the CA3 subfield. Many stained neurons were evident in the hilus, and some cells were also present in the subgranular zone and in the granular layer of the DG, in line with the distribution reported in adult animals (Deller and Leranth, 1990; Corvino et al., 2015) (Figures 3Aa–d). Unlike adult animals, in which TMT administration causes an increased expression of NPY-immunoreactivity (Corvino et al., 2015), both groups of TMT+oil-treated animals showed a reduction in NPY-IR cells in the CA1subfield (pyramidal layer and *stratum oriens*) (Figure 3Ae) and in the hilus (Figure 3Af), compared with CTRL-animals (Figures 3Aa–d), as indicated by stereological analysis followed by three-way ANOVA (Tukey *post hoc* test *p* < 0.05) (Figures 3Ba,b). Conversely, both groups of TMT+E2-treated animals exhibited levels of NPY-immunoreactivity similar to CTRL groups in the pyramidal layer of the CA1 subfield (Figures 3Ag, Ba) and in the hilus (Figures 3Ah, Bc) (three-way ANOVA; Tukey *post hoc* test *p* > 0.05). A reduction was still detectable in the CA1 *stratum oriens* (three-way ANOVA; Tukey *post hoc* test *p* < 0.05) (Figure 3Bb). No significant differences were found in the DG (Figure 3Bd) (three-way ANOVA; Tukey *post hoc* test *p* > 0.05). These findings were supported by qPCR analysis results, which showed a significant downregulation of the *npy* gene starting 3 days after intoxication in male TMT-treated animals (*p* < 0.05) (Figure 3Cc) and involving both groups 7 days after TMT treatment (*p* < 0.05) (Figures 3Cb,d). E2 administration was able to counteract this effect: expression levels of *npy* comparable to CTRL animals were detectable in the TMT+E2-treated groups (*p* > 0.05) at the later time point, which, in the F-TMT+E2 group, was even preceded by an earlier and transient upregulation (*p* < 0.05) (Figure 3C).

#### PV-Expressing Interneurons

**FIGURE 4:**
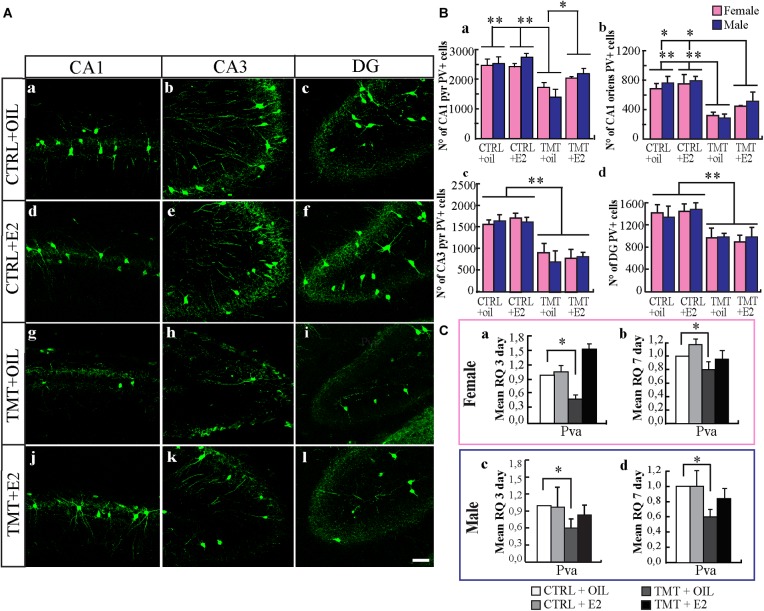
**(A)** Distribution of parvalbumin (PV)-immunostaining in the CA1, CA3 regions and dentate gyrus (DG) of the different experimental groups. Representative micrographs of PV-stained hippocampal sagittal sections from CA1 **(a,d,g,j)**, CA3 **(b,e,h,k)**, and DG **(c,f,i,l)** hippocampal regions of CTRL+oil- **(a–c)**, CTRL+17β-estradiol (E2)- **(d–f)**, TMT+oil- **(g–i)** and TMT+E2- **(j–l)** treated rats. A lower number of PV-IR cells is detectable in the CA1 and CA3 pyramidal layer of TMT+oil-treated rats compared with both control groups; conversely TMT+E2-treated rats exhibit a number of PV-IR cells comparable to those of CTRL+oil groups. Scale bar: 100 μm. **(B)** Numbers of PV-IR neurons in different hippocampal subfields of the different experimental groups. Stereological cell counts performed in the CA1 pyramidal layer, followed by three-way ANOVA revealed a significant effect of TMT treatment (*F*_1,29_ = 37.43 *p* < 0.001) and E2 administration (*F*_1,29_ = 6.95 *p* < 0.05); TMT^∗^E2 interaction was also significant (*F*_1,29_ = 4.53 *p* < 0.05). No significant difference related to sex was detectable (*F*_1,29_ = 0.26, *p* > 0.05). Tukey *post hoc* test showed a significant decrease in the number of PV-positive cells in the TMT+oil-treated groups (M-TMT+oil and F-TMT+oil) compared with CTRL groups (CTRL+oil and CTRL+E2) (*p* < 0.001) **(a)**. Both F- and M- TMT+E2-treated groups exhibited a number of PV-positive cells comparable to those of CTRL groups (Tukey *post hoc* test *p* > 0.05) and significantly higher than TMT+oil-treated groups (Tukey *post hoc* test *p* < 0.05) **(a)**. In the CA1 *stratum oriens* **(b)**, CA3 pyramidal layer **(c)**, and DG **(d)**, TMT administration induced an overall reduction of PV-immunoreactive cells, as pointed out by three-way ANOVA, which revealed a significant effect of TMT treatment (CA1 *stratum oriens*: TMT *F*_1,29_ = 55.17; CA3 pyramidal layer: TMT *F*_1,29_ = 52.69; DG: TMT *F*_1,29_ = 17.1; Tukey *post hoc* test *p* < 0.001); however, neither E2 administration (CA1 *stratum oriens*: TMT *F*_1,29_ = 3.02; CA3 pyramidal layer: E2 *F*_1,29_ = 0.13; DG: E2 *F*_1,29_ = 0.78; *p* > 0.05) nor sex (CA1 *stratum oriens*: TMT *F*_1,29_ = 0.73; CA3 pyramidal layer: sex *F*_1,29_ = 0.83 *p* > 0.05; DG: sex *F*_1,29_ = 0.178; *p* > 0.05) were significant. The values are given as means ± SE (^∗^*p* < 0.05, ^∗∗^*p* < 0.001). (F-CTRL+oil *n* = 6; M-CTRL+oil *n* = 5; F-CTRL+E2 *n* = 5; M-CTRL+E2 *n* = 4; F-TMT+oil *n* = 5; M-TMT+oil *n* = 4; F-TMT+E2 *n* = 4; M-TMT+E2 *n* = 4). **(C)** Expression levels of *parvalbumin (pva)* gene and its modulation in the hippocampus of TMT-treated rats by E2 administration. Bar graphs represent results of quantitative real-time PCR obtained using the ΔΔCt method for the calculation of relative quantity (RQ) of *pva gene*, 3 **(a,c)** and 7 **(b,d)** days after TMT administration both in female **(a,b)** and male **(c,d)** rats; ^∗^*p* < 0.05, calculated on mean ΔCt across biological replicates. TMT administration is associated with a significant and early occurring downregulation of *pva* gene both in males and females, which is still detectable 7 days after intoxication (M-TMT+oil vs. M-CTRL oil and F-TMT+oil vs. F-CTRL+oil: *p* < 0.05). On the contrary, TMT+E2-treated animals show *pva* expression levels comparable to those of CTRL animals at the two time points explored (M-TMT+oil vs. M-CTRL oil and F-TMT+oil vs. F-CTRL+oil: *p* > 0.05) (*n* = 4/each experimental group).

The distribution of PV-immunoreactivity reflected that previously reported in young rodents (Dell’Anna et al., 1996; Geloso et al., 1998). In CTRL animals, PV-IR cells were present in all hippocampal layers, mainly localized in the *stratum oriens* and pyramidal cell layer of the CA and in the granule cell layer of the DG. A significant lower number of PV-positive cells was evident in the TMT+oil-treated groups (Figures 4Ag–i) compared with both CTRL groups (Figures 4Aa–f) in all hippocampal subfields considered, namely CA1 *stratum oriens*, CA1 pyramidal layer, CA3 pyramidal layer and DG (*p* < 0.05). Notably, stereological cell counts indicated that the number of PV-IR neurons in both TMT+E2-treated groups was significantly higher than that in the TMT+oil-treated animals (three-way ANOVA; Tukey *post hoc* test *p* < 0.05) and was also comparable to that of CTRL animals in the CA1 pyramidal layer (Figure 4Ba). On the contrary, a reduction was still present in both groups at the level of the CA1 *stratum oriens*, in the CA3 pyramidal layer and in the DG (three-way ANOVA; Tukey *post hoc* test *p* < 0.05) (Figures 4Bb–d).

Consistently, qPCR analysis of *pva* gene expression revealed that TMT administration was associated with a significant and early-occurring downregulation of *pva* gene in both female and male rats (Figures 4Ca,c), which is still detectable 7 days after intoxication (*p* < 0.05) (Figures 4Cb,d). On the contrary, TMT+E2-treated animals showed *pva* expression levels comparable to those of CTRL animals at the two time points investigated (*p* > 0.05) (Figure 4C).

PNNs, structures made by extracellular matrix proteins and proteoglycans enveloping GABAergic interneurons, are deeply involved in the functional maturation of PV-interneurons (Cabungcal et al., 2013; Umemori et al., 2015) and contribute to critical-period closure (Pizzorusso et al., 2002). In particular, one of their components, namely AG, is found mainly around PV-expressing interneurons (McRae et al., 2010). In order to explore possible impairment of PNN caused by TMT intoxication, which may be involved in the TMT-induced reduction of PV immunoreactivity, we evaluated possible variations in the percentage of double-labeled PV/AG cells in the hippocampi of all experimental groups.

**FIGURE 5:**
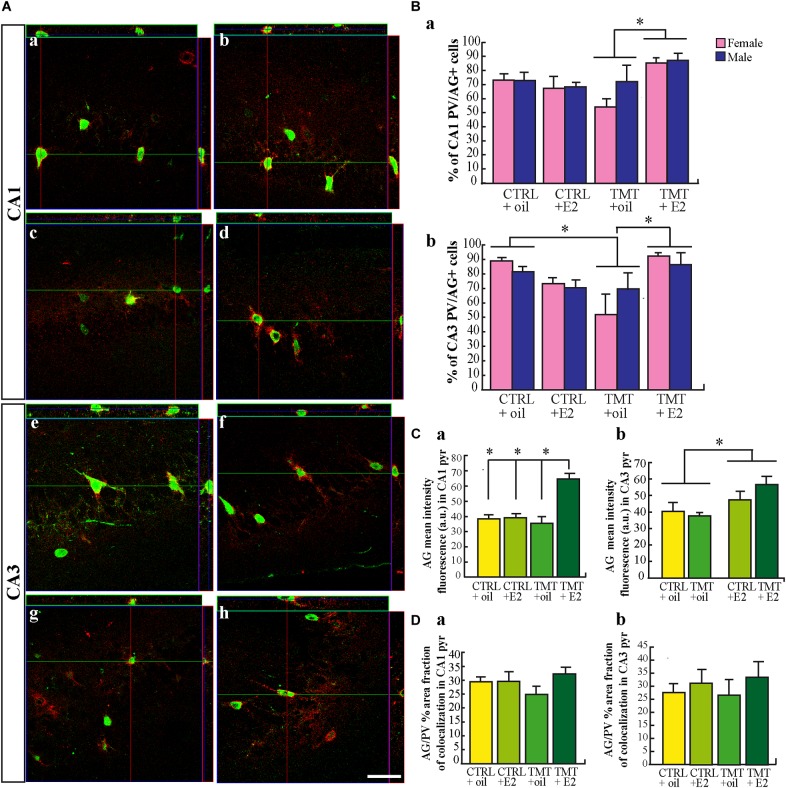
Aggrecan (AG) expression in the hippocampi of the different experimental groups. **(A)** Representative confocal reconstructed orthogonal images, as viewed in the x-z (top) and y-z (right) planes of 40 μm sagittal sections from CA1 **(a–d)** and CA3 **(e–h)** hippocampal subfields of CTRL+oil- **(a,e)** CTRL+17β-estradiol (E2)- **(b,f)**, TMT+oil- **(c,g)**, TMT+E2- **(d,h)** treated rats, double-labeled for parvalbumin (PV) (green) and AG (red). Scale bar: 90 μm in **(a–d)**, 100 μm in **(e-h)**. **(B)** Percentage of PV/AG-positive neurons in the CA1 and CA3 pyramidal layer of CTRL+oil-, CTRL+E2-, TMT+oil-, TMT+E2- treated rats. In the CA1 region quantitative analysis followed by three-way ANOVA indicates a significant effect of both E2 administration (*F*_1,18_ = 4.42 *p* < 0.001) and TMT^∗^E2 interaction (*F*_1,18_ = 13.9, *p* < 0.05), while no significant difference related to sex emerged (*F*_1,18_ = 1.5, *p* > 0.05). TMT+E2-treated rats (M-TMT+E2 and F-TMT+E2) exhibited a significantly higher percentage of PV/AG-double labeled interneurons compared with TMT+oil groups (M-TMT+oil and F-TMT+oil; Tukey *post hoc p* < 0.05). No differences related to sex are present (*F*_1,18_ = 0.15, *p* > 0.05) **(a)**. Statistical analysis performed in CA3 pyramidal layer **(b)** showed a significant TMT^∗^E2 interaction (three-way ANOVA *F*_1,18_ = 11.62, *p* < 0.05), pointing out a reduced percentage of PV/AG co-localization in TMT+oil rats (M-TMT+oil and F-TMT+oil) compared with CTRL+oil groups (F-CTRL+oil and M-CTRL+oil, Tukey *post hoc p* < 0.05), while the TMT+E2-treated group showed a percentage of PV/AG co-localization significantly higher than TMT+oil groups (Tukey *post hoc p* < 0.05) and similar to that in CTRL groups (Tukey *post hoc p* > 0.05). No differences related to sex are present (*F*_1,18_ = 0.01, *p* > 0.05). The values are given as means ± SE (^∗^*p* < 0.05). (F-CTRL+oil *n* = 3; M-CTRL+oil *n* = 3; F-CTRL+E2 *n* = 4; M-CTRL+E2 *n* = 3; F-TMT+oil *n* = 4; M-TMT+oil *n* = 3; F-TMT+E2 *n* = 3; M-TMT+E2 *n* = 3). **(C)** Mean fluorescence intensity of PV/AG stained cells in the hippocampi of the different experimental groups. Bar graphs indicate the mean fluorescence intensity of PV/AG+ neurons in both CA1 **(a)** and CA3 **(b)** hippocampal subfields of CTRL+oil-, CTRL+E2-, TMT+oil-, TMT+E2- treated animals. In the CA1 subfield (pyramidal layer), two-way ANOVA showed a significant effect of both TMT treatment (*F*_1,20_ = 8.18, *p* < 0.05) and E2 administration (*F*_1,20_ = 18.16, *p* < 0.001); TMT^∗^E2 interaction (*F*_1,20_ = 16.02; *p* < 0.001) was also significant. Tukey *post hoc* test showed a significant increase in the mean fluorescence intensity of AG-positive cells in the TMT+E2-treated groups compared with all other experimental groups (*p* < 0.001, for all comparisons) **(a)**. In the CA3 region, only the significant effect of E2 administration was detectable (two-way ANOVA, *F*_1,20_ = 9.72, *p* < 0.05 Tukey *post hoc p* < 0.05) thus indicating an increased mean fluorescence intensity of AG in both groups of E2-treated animals **(b)**. The values are given as means of pooled sex groups (male + female) ± SE both in **(C,D)** (^∗^*p* < 0.05, ^∗∗^*p* < 0.001) (*n* = 6 animals/each experimental group). **(D)** Quantitative analysis of percentage of fraction area in PV/AG-stained cells. No significant differences in the percentage of fraction area emerged among the different experimental groups both in the CA1 **(a)** and CA3 **(b)** subfields (two-way ANOVA, *p* > 0.05). The values are given as means of pooled sex groups (male + female) ± SE both in **(C,D)** (^∗^*p* < 0.05, ^∗∗^*p* < 0.001) (*n* = 6 animals/each experimental group).

A slight and not significant decrease in the percentage of double-stained PV/AG cells was found in the CA1 subfield of TMT+oil-treated animals (three-way ANOVA; Tukey *post hoc p* > 0.05; Figures 5Aa–d, Ba), while a significant reduction in the percentage of double-stained PV/AG was present in the CA3 region (Figures 5Ae–h, Bb) (three-way ANOVA; Tukey *post hoc p* < 0.05) (Figure 5Bb). This suggests that a degradation of the PNN occurs in this region after TMT administration. In contrast, both groups of TMT+E2-treated rats showed levels of co-localization similar to CTRL rats and significantly higher than TMT+oil-treated animals (three-way ANOVA; Tukey *post hoc p* > 0.05) (Figure 5B).

Mean fluorescence intensity and percentage of fraction area were also analyzed. In the CA1 subfield a significant increase in the mean fluorescence intensity of AG-positive cells emerged in the TMT+E2-treated groups compared with all other experimental groups (two-way ANOVA; Tukey *post hoc p* < 0.001, for all comparisons) (Figure 5Ca). In the CA3 region, only the significant effect of E2 administration was detectable, indicating an increased mean fluorescence intensity of AG in both groups of E2-treated animals in this subfield (two-way ANOVA; Tukey *post hoc p* < 0.05) (Figure 5Cb).

No significant differences in the percentage of fraction area emerged among the different experimental groups in either the CA1 (Figure 5Da) or CA3 (Figure 5Db) subfields (two-way ANOVA, *p* > 0.05).

## Discussion

While in adult experimental models of brain disease the neuroprotective properties of E2 are widely documented (Garcia-Segura et al., 2001; Amantea et al., 2005; Brann et al., 2007; Brinton, 2009; Arevalo et al., 2015), its effects in counteracting brain damage during postnatal development still deserve further investigation (McCarthy, 2008).

In this regard, the present study shows that the pretreatment with E2 exerts a protective effect against hippocampal injury induced by TMT administration early in development. Our findings show that E2 administration reduces both the extent of TMT-induced neuronal death in the CA1 subfield, consistently with the regional specificity which characterizes E2-mediated effects at the hippocampal level (Woolley, 2000; Dai et al., 2007; McCarthy, 2009), and the impairment of PV- and NPY- expression induced by the toxicant in selected hippocampal regions.

Consistently with our previous observation in TMT-treated adult animals (Corvino et al., 2015), we found that E2 administration is able to stimulate the activation of endogenous neuroprotective pathways in the TMT-injured hippocampus. In particular, exogenous E2 induces a long-lasting upregulation of *bcl-2* in TMT-treated rat pups, which overcomes the downregulation induced by TMT administration, in line with the notion that estrogens regulate apoptosis by enhancing the expression of the anti-apoptotic protein Bcl-2 (Wu et al., 2005; McCarthy, 2008, 2009). Treatment with E2 also induces a delayed upregulation of *bcl-2* expression in the hippocampi of control female rat pups. It is known that, by regulating apoptosis, Bcl-2 exerts a modulatory role on developmental processes in the brain (Opferman and Kothari, 2018). Interestingly, it has been reported that high postnatal neuronal expression of *bcl-2* is selectively clustered in regions characterized by postnatal neurogenesis, such as the hippocampus (Merry et al., 1994). Since full maturation of the DG occurs postnatally, we may speculate that the administration of E2 may trigger events that influence this process in control female rats.

In addition, our analysis pointed out some gender-related differences in the changes induced by TMT in *bcl-2* expression. Our finding is in line with the notion that sex-differences in signaling pathways involved in neuronal death are present in both the adult and the neonatal brain (Lang and McCullough, 2008). In particular, similarly to the present experimental conditions, differences in the molecular machinery leading to apoptosis have been described in other models of neonatal brain damage, in spite of similar extent of brain injury (Zhu et al., 2006). Estrogens, through ER-α, are also known to modulate the BDNF and Trkb pathways at the hippocampal level (Scharfman and Maclusky, 2005). For this reason, we explored the expression pattern of *bdnf* and *trkb* in the different experimental conditions. Differently from adult animals (Corvino et al., 2015), we did not detect any modulation of the *bdnf* gene or its receptor *trkb* after TMT administration in rat pups. We may hypothesize that age-related differences in the activation of this molecular pathway, as well as differences in the time frames of activation could explain this discrepancy. In contrast, we found an early and persistent upregulation of *trkB* in TMT-treated animals after pretreatment with E2, in absence of *bdnf* modulation. This is consistent with recent data indicating that the modulation of Trkb-dependent pathways plays a relevant role in E2-related neuroprotective effects during development (Cikla et al., 2016). Many studies point out that transactivation of TrkB may be induced by molecules different from neurotrophines (Lee and Chao, 2001; Huang et al., 2008), including E2 through non-classical ER (Briz and Baudry, 2014), therefore a mechanism of TrkB modulation independent from BDNF could be responsible for the reported observation.

In accordance with previous findings in adult animals (Corvino et al., 2013; Lattanzi et al., 2013), TMT-induced neuronal death in the neonatal hippocampus is associated with early and marked neuroinflammatory changes. Interestingly, our data indicate that pretreatment with E2 induces, in both males and females, a reduction in the inflammatory background underlying TMT-induced neuronal death, by reducing the amount of activated microglia, supporting the early upregulation of the protective microglial phenotype, and decreasing the expression of the astroglial cytokine S100B, which acts, at high concentrations, as a proinflammatory agent (Adami et al., 2004; Bianchi et al., 2007; Michetti et al., 2018). Inhibition of the neuroinflammatory cascade is emerging as one of the most relevant mechanisms involved in E2-mediated neuroprotection (Pansiot et al., 2016, 2017; Slowik et al., 2017). The wide expression of both ERα and ERβ in non-neuronal cells of the CNS, including microglia, is believed to underlie its possible, direct modulatory role on microglial activation (Slowik et al., 2017) and polarization (Siani et al., 2017).

Sex-dependent differences in the expression profile of some proinflammatory cytokines emerged, in line with studies highlighting the sexual dimorphism of microglia during postnatal development that involves also mechanisms related to microglial activation (Schwarz et al., 2012; Mallard et al., 2018). This may cause discrepancies between sexes in the molecular pathways driving neuroinflammation (Mallard et al., 2018), that may explain our results.

Together with the activation of anti-apoptotic and neurotrophic pathways, this scenario highlights the role exerted by E2 in the fine-tuning of brain-intrinsic reactive responses to injuries.

In the developing brain, E2 is also a strong modulator of synaptogenesis and plasticity (McCarthy, 2008, 2009), and these properties are involved in its neuroprotective effects (Wartman et al., 2012). As in the adult brain, in the developing hippocampus E2 influences the GABAergic system (Pytel et al., 2007; McCarthy, 2008; Wójtowicz and Mozrzymas, 2010) and this effect is also mediated by its interaction with selected interneuron subpopulations expressing estrogen receptors, such as PV- and NPY-IR cells (Blurton-Jones and Tuszynski, 2006; Nakamura et al., 2007; Higaki et al., 2012; Tibrewal et al., 2018).

Stressful events in early life can affect inhibitory circuits, at both the structural and neurochemical levels. Accordingly, TMT-induced hippocampal damage during postnatal development is associated with a reduced expression of PV, which, in the present conditions, also involves the CA1 subfield and the DG, in contrast with our earlier findings (Geloso et al., 1996, 1998). This discrepancy is not surprising and may be related to the different experimental design of the present study, in which a higher dose of TMT was administered at later time point, when the hippocampus is more susceptible to the toxicant.

PV-IR interneurons are characterized by postnatal maturation and PV becomes detectable in hippocampal neurons approximately at the same time (P4–P8) as TMT administration (Bergmann et al., 1991; Solbach and Celio, 1991; Alcantara et al., 1993). Since they are particularly susceptible to different *noxae* during development, including oxidative stress (Jiang et al., 2013) and neuroinflammation (Jenkins et al., 2009), which are both hallmarks of TMT-induced neurodegeneration (Geloso et al., 2011), they may express a particular vulnerability to the toxicant in this period (Geloso et al., 1998). On the other hand, the reduced number of PV-IR neurons might also be the consequence of a downregulation of the protein, as hypothesized in other models (Filice et al., 2016), thus suggesting either a delay in the maturation of these interneurons induced by TMT administration, or, alternatively, their functional impairment. This hypothesis could fit with the impaired PV-immunoreactivity reported at the level of the DG, where no degenerating neurons were detectable.

Changes in cell numbers or defective maturation could result in persistent functional alterations at distant time points (Fine et al., 2014). In particular, dysfunction or defective maturation of PV-positive interneurons has been proposed as a possible causative factors in neuropsychiatric and neurodevelopmental disorders (Jiang et al., 2013; Fine et al., 2014; Ito-Ishida et al., 2015; Wöhr et al., 2015; Filice et al., 2016). For this reason, our findings may be relevant from a translational perspective, and, on this basis, TMT-induced hippocampal damage during development could represent a useful experimental model to study possible pharmacological approaches aimed at modulating this interneuron subpopulation. In the light of this, it is remarkable that E2 administration improves PV expression in the pyramidal layer of the CA1 region. It could be a consequence of the milder TMT-induced hippocampal damage in this region. On the other hand, growing evidence supports a modulatory role of E2 on PV expression in both non-neural (Aboghe et al., 2009) and neural tissues (Macrì et al., 2010; Koh, 2014; Wu et al., 2014, 2015; Corvino et al., 2015; Filice et al., 2018). At the hippocampal level, this effect seems to be particularly evident in the dorsal CA1 hippocampal subfield (Wu et al., 2015). For this reason, a direct role of E2 administration on PV expression cannot be ruled out.

The functional properties of PV-IR cells are strictly related to PNN, a specialized structure of the extracellular matrix most prominently displayed around GABAergic interneurons (McRae and Porter, 2012). PNN appears during postnatal development (McRae et al., 2010; Horii-Hayashi et al., 2015; Lensjø et al., 2017), is a plastic structure, that is sensitive to environmental changes (McRae et al., 2010), and plays a role in the remodeling of inhibitory network (Castillo-Gómez et al., 2017). Although an altered expression of PNN has not been reported in adult rats after TMT-intoxication (Schüppel et al., 2002), our data suggest that a degradation of PNN occurs in the CA3 region after TMT administration. It should be noted that at P7 the immature and less condensed PNN could be more vulnerable to external *noxae* (Cabungcal et al., 2013; Horii-Hayashi et al., 2015). Therefore, also taking into account the protective role played by this structure against oxidative stress (Cabungcal et al., 2013), we may speculate that damage to immature PNN could play a relevant role in the loss of PV-expressing interneurons reported in the CA3 region, where no recovery of PV-immunoreactivity was observed after E2 treatment. On the contrary, E2 administration increases AG expression in the TMT-injured hippocampus. E2 has been shown to directly promote the synthesis of extracellular matrix proteins including AG (Li et al., 2017) and we may therefore speculate that this mechanism could contribute to the protective role on PV expression in interneurons, albeit restricted to the CA1 region.

Our data indicate that TMT-induced neurodegeneration early in development is also accompanied by a reduction in NPY expression. It is known that NPY-IR interneurons exhibit a dynamic pattern of maturation during the postnatal period and NPY-immunoreactivity reaches adult levels at P21 (Soriano et al., 1994; Moryś, 2002). Also in this case, we may hypothesize that they could be particularly vulnerable in this early period of development to the toxic action of TMT, in contrast to our previous observation in the adult hippocampus (Corvino et al., 2015). The reduced number of NPY-IR cells could therefore be related to cell loss. On the other hand, NPY-expression is known to be induced by neuronal activity (Marty, 2000) and it has been proposed that it could be “latent” in various types of interneurons (Vitalis and Rossier, 2011). For this reason, the reduced number of NPY-IR neurons could also be related to a reduced expression of the peptide.

Many findings suggest that E2 is able to induce increased NPY synthesis in hippocampal neurons (Scharfman and Maclusky, 2006), especially in the hilus (Velísková and Velísek, 2007; Corvino et al., 2015) and in the CA1 subfield (Nakamura and McEwen, 2005; Corvino et al., 2015). In line with this notion, after E2 administration, both groups of TMT-treated animals exhibited levels of NPY-immunoreactivity comparable to those of CTRL animals in those regions. Notably, reduced expression of NPY in the hilus is believed to impair the functional activity of the DG (Freund and Buzsaki, 1996; Hsu, 2007), while enhanced expression of the peptide in the hilar region has been associated with neuroprotective effects (Velísková and Velísek, 2007; Ledoux et al., 2009).

Together with the observed restoration of PV immunoreactivity in the same region, recovered NPY expression may be part of a compensatory mechanism triggered by E2 administration, possibly aimed at establishing a correct excitatory/inhibitory balance in the TMT-injured hippocampus. In addition, increased expression levels of the *npy* gene, even higher than control rats were detectable in female rats, both in CTRL+E2- and TMT+E2-treated animals. Sex-related differences in NPY expression under different experimental conditions have been described (Mazid et al., 2016; Karisetty et al., 2017) and may explain our observation.

It should be noted, however, that the reduction in both PV- and NPY- expressing interneurons induced by TMT administration in the CA1 *stratum oriens*, where no degenerating neurons were found, was not influenced by pretreatment with E2. The complex interneuron subpopulations of the *stratum oriens*, among which those expressing PV and NPY are a subgroup, are deeply involved in the CA1 microcircuit (Lacaille et al., 1987). Since they are specialized to modulate the excitatory drive coming from the main input regions to CA1, including CA3 (Müller and Remy, 2014), it is possible that the reduced expression of both PV and NPY could reflect an alteration of the hippocampal circuitry that cannot be reversed by the compensatory phenomena triggered by E2 administration.

Although restricted to the pyramidal cell layer of the CA1 region, our data support the efficacy of pretreatment with E2 in reducing the extent of TMT-induced hippocampal damage in developing rats. On the other hand, it should be taken into consideration that early-life exposure to estrogens, even in single dose, may negatively impact the development of the reproductive system in the long term, both in males (Atanassova et al., 2005; Vrooman et al., 2015), and in females (Ikeda et al., 2001; Takahashi et al., 2013).

Additional studies are needed to assess the extent of functional protection as well as possible long-term side effects, to validate the translational potentials of estrogen-based neuroprotective approaches aimed at counteracting early hippocampal damage.

## Ethics Statement

All animal procedures were approved by the Ethic Committee of the Catholic University (authorization number 100/2016-PR) and were fully compliant with the Italian Ministry of Health guidelines (Legislative Decree No. 26/2014).

## Author Contributions

EM participated to the conception of the experiments, performed the experiments, wrote part of the manuscript, edited the manuscript, and revised it critically. VC provided a substantial contribution to the conception and design of experiments, performed the experiments, analyzed the data, wrote part of the manuscript, and critically revised it. VDM, AF, SG, PL, and EC performed part of the experiments. FM provided substantial financial support to the research and critically revised the manuscript. MCG conceived the work and contributed to the financial support, gave substantial contributions to the design of the experiments, to the analysis and interpretation of data, drafted the work, and revised it critically.

## Conflict of Interest Statement

The authors declare that the research was conducted in the absence of any commercial or financial relationships that could be construed as a potential conflict of interest.

## Footnotes

1 http://primer3.sourceforge.net/
