# Supplementary material for: The Neuroprotective Effects of 17β-Estradiol Pretreatment in a Model of Neonatal Hippocampal Injury Induced by Trimethyltin
Source: Front Cell Neurosci. 2018 Oct 26;12:385. doi: 10.3389/fncel.2018.00385 (PMC6213803; doi:10.3389/fncel.2018.00385)
Supplement: Supplementary file 1 [file Table_1.docx]

***Supplementary Material***

**The neuroprotective effects of 17**β **-estradiol pre-treatment in a model of neonatal hippocampal injury induced by trimethyltin.**

Elisa Marchese, Valentina Corvino, Valentina Di Maria, Alfredo Furno, Stefano Giannetti, Eleonora Cesari, Paola Lulli, Fabrizio Michetti and Maria Concetta Geloso^*^

* Correspondence: Maria Concetta Geloso

Institute of Anatomy and Cell Biology

Università Cattolica del Sacro Cuore

Largo Francesco Vito 1, 00168 Rome, Italy

tel: +390630154915

Fax: +39 06 30154813

e-mail: [mariaconcetta.geloso@unicatt.it](mailto:mariaconcetta.geloso@unicatt.it)

# Supplementary Figures and Tables

## Supplementary Figures


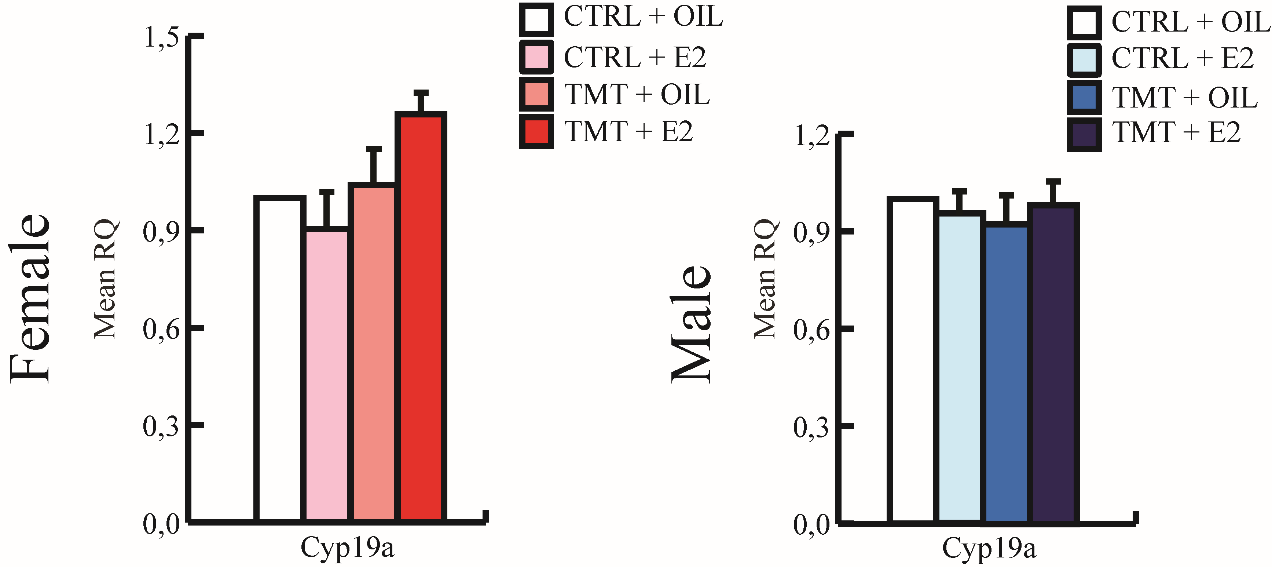


**Supplementary Fig. 1. Expression levels of Aromatase (*Cyp19a*) in the hippocampi of the different experimental groups.** Bar graphs represent results of quantitative real time-PCR obtained using the ΔΔCt method for the calculation of relative quantity (RQ) of *Cyp19a*. No significant differences in the expression levels of *Cyp19a* were detectable among groups (p>0.05). The values are given as means of pooled sex groups (male+female) ±S.E.


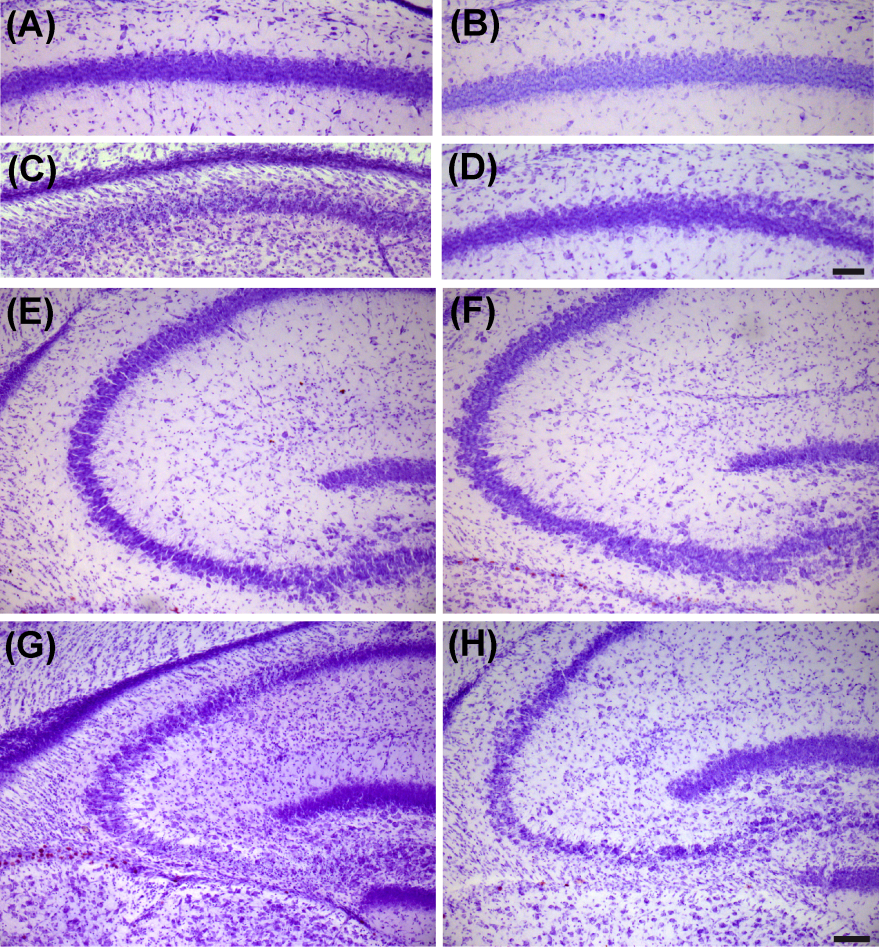


**Supplementary Fig.** **2**. **Extent and features of TMT-induced hippocampal damage in developing rats**. A-H: Representative micrographs of Nissl-stained hippocampal sagittal sections from CA1 (A-D) and CA3 subfields (E-H) of CTRL+oil- (A, E), CTRL+E2- (B, F), TMT+oil- (C, G), TMT+E2- (D, H) treated rat pups. Neuronal loss is clearly detectable in CA3 and CA1 pyramidal neurons of both TMT+oil- (C, G) and TMT+E2-(D, H) treated animals. Scale bar 150 µm.

## Supplementary Tables

**Supplementary Table 1**: Oligonucleotide primer sequences.

| **Gene Symbol** | **Forward Primer** | **Reverse Primer** |
| --- | --- | --- |
| **Bcl-2** | 5’-tcaaagaaggccacaatcct-3’ | 5′-ggtggtggaggaactcttca-3’ |
| **Bdnf** | 5’-cgaaccttctggtcctcatc-3’ | 5’-tggctgacacttttgagcac-3’ |
| **Ntrk2** | 5’-caagctgacgagtttgtcca-3’ | 5’-ttacccgtcaggatcaggtc-3’ |
| **Pva** | 5’-cgaccacaaaaagttcttcca-3’ | 5’-ccgcactctttttctcagg-3’ |
| **Npy** | 5’-tactccgctctgcgacacta-3’ | 5’-tctcagggctggatctcttg-3’ |
| **Il1b** | 5’-catcagcacctctcaagcag-3’ | 5’-aactatgtcccgaccattgc-3’ |
| **Tnf** | 5’-cagcagatgggctgtacctt-3’ | 5’-gtgggtgaggagcacgtagt-3’ |
| **Il6** | 5’-accccaacttccaatgctct-3’ | 5’-tggtccttagccactccttc-3’ |
| **Chi3l3** | 5’-ctgaatgatggagccactga-3’ | 5’-aacccatacattgccctgaa-3’ |
| **S100b** | 5’-gtggtggacaaagtgatgga-3’ | 5’-ggaagtcacactccccatcc-3’ |
| **Cyp19a1** | 5’- ctcctcctgattcggaattgt-3’ | 5’-tctgccatgggaaatgagag-3’ |
| **Il10** | 5’-ctgctgacagattccttactgc-3’ | 5’-cctggggcatcacttctacc-3’ |
| **Il4** | 5’-actccatgcaccgagatgtt-3’ | 5’-agatgagctcgttctccgtg-3’ |
| **Actb** | 5’-gacccagatcatgtttgagacct-3’ | 5’-accagaggcatacagggaca-3’ |
